# Antiretroviral therapy adherence monitoring and its impact on immuno-virological outcome

**DOI:** 10.1186/1471-2334-14-S4-O8

**Published:** 2014-05-29

**Authors:** Alina Lobodan, Cristina Popescu, Anca Ruxandra Negru, Raluca Dulamă, Irina Lăpădat, Violeta Molagic, Mihaela Rădulescu, Raluca Jipa, Adriana Hristea, Raluca Mihăilescu, Cătălin Tilişcan, Daniela Munteanu, Raluca Năstase, Gabriel Popescu, Victoria Aramă

**Affiliations:** 1 National Institute for Infectious Diseases “Prof. Dr. Matei Balş”, Bucharest, Romania

## 

One of the most important factors in achieving a good outcome is treatment adherence. Poor adherence to antiretroviral therapy (ART) leads to less viral suppression, permanent treatment resistance and increased costs. There are multiple causes of poor adherence: regimen complexity, side effects etc.

Aim: to analyze ART adherence, risk factors for poor adherence and their impact on the outcome.

We performed a one year survey (from January to December 2013) of HIV infected patients monitored in the Third Department of the National Institute for Infectious Diseases “Prof. Dr. Matei Balş”. The data (number of days covered by ART) was collected from patients’ files. We correlated the adherence with gender, regimen rank and complexity. Statistical analysis was made using EPI INFO 6.

We retrospectively analyzed 111 patients who came in to our clinic monthly to pick-up their ART, 52 women (46.84%) and 59 men (53.16%) with a mean age of 43.5 years old. The adherence to ART was: 23 (20.62%) – 100% adherence, 36 (32.43%) – more than 96.7% adherence (less than 12 days without medication), 38 (34.23%) – 91.8% to 96.7% adherence (13-30 days without medication), 10 (9%) – 83.6% to 91.8% adherence (30-60 days without medication) and 4 (3.6%) with less than 83.6% adherence (more than 60 days without medication). The level of adherence was correlated with therapeutic failure: for 100% adherence – two failures (8.69%), for more than 96.7% adherence – no failure, for more than 91.8% adherence – 4 failures (11.1%), for more than 83.6% adherence – 2 failures (20%) and for less than 80% adherence – 3 failures (75%). Adherence below 91.8% was correlated with treatment failure: RR 5.65 (CI95% 1.99; 16.09, p=0.0007). We analyzed some possible risk factors for poor adherence: gender, regimen rank and complexity. Although 51.95% from the non-adherence group were women, the adherence wasn’t correlated with gender: RR 1.23 (CI95% 0.93; 1.62, p=0.16). A regimen rank higher than 1 was correlated with low adherence – 45.76% vs. 28.84% in the adherence vs. non-adherence group: RR 1.5 (CI95% 0.95; 2.38, p=0.07). The regimen containing protease inhibitors wasn’t correlated with low adherence: 33.9% vs. 30.8%, RR 1.08 (CI95% 0.71; 1.67, p=0.73).

We emphasize the impact of therapy adherence on the outcome. A level of adherence below 91% was correlated with therapeutic failure. ART adherence wasn’t correlated with gender, PI regimen and rank regimen.

